# Silent Threat Evolution: Critically Important Carbapenem and Colistin Resistance Genes in the Natural Aquatic Environment

**DOI:** 10.3390/antibiotics15020113

**Published:** 2026-01-23

**Authors:** Małgorzata Czatzkowska, Damian Rolbiecki

**Affiliations:** 1 Department of Water Protection Engineering and Environmental Microbiology, Faculty of Geoengineering, University of Warmia and Mazury in Olsztyn, 10-720 Olsztyn, Poland; malgorzata.czatzkowska@uwm.edu.pl; 2 European Regional Centre for Ecohydrology of the Polish Academy of Sciences, 90-364 Lodz, Poland

**Keywords:** natural aquatic environment, carbapenem resistance, mobile colistin resistance, last-line drugs, antimicrobial resistance, clinical importance

## Abstract

The rise in antimicrobial resistance (AMR) among the most clinically significant bacteria presents a global threat. The coexistence of resistance mechanisms to both carbapenems and colistin is particularly concerning, as these are last-line treatments, specifically reserved for the most challenging infections caused by clinically multidrug-resistant Enterobacterales. Natural aquatic environments have become environmental reservoirs for the transmission of AMR, particularly concerning mechanisms against these two types of critically important drugs. The crucial role of environmental settings as a driving force for the spread and evolution of AMR associated with these drugs is underestimated, and scientific knowledge on this topic is limited. This review aims to fill an important gap in the scientific literature and comprehensively consolidate the available data on carbapenem- and colistin-associated AMR in the aquatic environment. This study provides a comprehensive synthesis of the current knowledge by integrating bibliographic data with a detailed genomic analysis of 278 bacterial genomes sourced from natural waters. It explores the distribution of carbapenemase and mobile colistin resistance (*mcr*) genes, identifying their hosts, geographical spread, and complex gene–plasmid–host associations. This review distinguishes two critical host groups for genes that provide resistance to last-resort drugs, Enterobacterales and autochthonous aquatic microbiota, highlighting both confirmed and potential interactions between them. Crucially, genomic analysis highlights the alarming co-occurrence of carbapenem and colistin resistance in single cells and on single plasmids, contributing to the spread of multidrug resistance phenotypes. These findings clearly indicate that aquatic environments are not merely passive recipients but active, evolving hubs for high-risk AMR determinants. Future research should focus on the interplay between allochthonous vectors and autochthonous microbiota to better understand the long-term stabilization of carbapenemase and *mcr* genes. Such efforts, combined with advanced sequencing technologies, are essential to ensure that carbapenems and colistin remain viable treatment options in clinical settings.

## 1. Introduction

Antimicrobial resistance (AMR) is a significant global health issue resulting from the unavoidable consequence of the misuse of antibiotics in animal and human health, as well as in agriculture [1]. The increasing use of antimicrobial drugs contributes to the spread of antibiotic-resistant bacteria (ARB) and antibiotic resistance genes (ARGs), creating a “silent pandemic” [2]. The widespread prevalence of ARB and ARGs has severely undermined the significant advancements made in antibacterial therapy, causing common infections to be untreatable [3]. At present, estimates suggest that deaths from infections caused by resistant organisms could reach 10 million by 2050 [4]. Currently, AMR is recognized as one of the most pressing health challenges worldwide, driving us toward a post-antibiotic era [1,5,6]. The most significant consequence of AMR is the reduced effectiveness of many antimicrobials, leaving only a limited number of last-resort options for treating infections. The high rates of AMR observed among the most clinically important bacteria that cause common infections pose a major problem [4,7].

On 17 May 2024, the World Health Organization (WHO) released an updated list of ARB that pose the most significant threat to human health [8]. This update aims to guide the development of new treatments and strategies to prevent the spread of AMR [9]. The list of priority pathogens includes members of the Enterobacterales order that are resistant to carbapenems and produce extended-spectrum β-lactamases (ESBLs), which are of particular concern in hospital epidemiology [10]. Carbapenems, belonging to β-lactam antibiotics, present a broad spectrum of antibacterial activity. These antimicrobial substances are highly effective against both Gram-positive and Gram-negative bacteria [11,12]. Carbapenems work primarily by inhibiting the synthesis of peptidoglycan, a crucial component of bacterial cell walls. The unique molecular structure of antimicrobial substances is due to the presence of a carbapenem together with the β-lactam ring, which confers exceptional stability against most β-lactamases, including ESBLs [12]. These antibiotics are frequently used in human medicine to treat infections caused by various bacteria, including *Escherichia coli*, *Salmonella* sp., *Klebsiella pneumoniae*, *Pseudomonas aeruginosa*, *Mycobacterium tuberculosis*, and methicillin-resistant *Staphylococcus aureus* (MRSA). Their safety profile has contributed to their widespread use across all continents, which has unfortunately led to an increase in carbapenem resistance among microorganisms [11].

Bacteria develop carbapenem resistance by either producing carbapenemase, an enzyme that degrades the carbapenem, or acquiring structural mutations that induce the production of other β-lactamases [13,14]. The existence of carbapenemases has been recognized for over 30 years. The IMP-1 was the first carbapenemase detected in Japan in 1991 [15]. A few years later, in Italy, VIM-1 was reported [16]. The genes responsible for producing these enzymes, namely *bla*_IMP_ and *bla*_VIM_, have successfully spread worldwide among microorganisms. Currently, the most common carbapenemases recognized among bacteria include *Klebsiella pneumoniae* carbapenemase (KPC), metallo-β-lactamases (MBLs), New Delhi metallo-β-lactamase (NDM), and oxacillinases (OXA) [14]. These enzymes allow bacteria to become resistant not only to carbapenems but also to many other β-lactam antibiotics, such as cephalosporins, monobactams, and penicillins [17]. In contrast to the sporadic reports of GIM, SIM, AIM, DIM, FIM, and POM carbapenemases, NDM has shown a dramatic global spread since its detection in India in 2008 [18]. In turn, among the class A carbapenem-hydrolyzing enzymes, KPCs have exhibited the most rapid and widespread dissemination [12]. The genes encoding these enzymes, *bla*_NDM_ and *bla*_KPC_, respectively, are widely distributed both in clinical and non-clinical settings [19].

The broad use of carbapenems in clinical practice and the potential to transfer their ARGs through mobile genetic elements (MGEs) have promoted the emergence of carbapenem-resistant bacteria, which include species of particular clinical importance. Especially the pathogenic species belonging to *Enterobacteriaceae* are commonly associated with hospital infections such as pneumonia, bacteriemia, or urinary tract infections [20,21]. Carbapenems, which display a broad spectrum of activity, are considered a last-line drug against infections caused by multidrug-resistant (MDR) bacteria belonging to this family [22]. However, due to the spread of carbapenem resistance among *Enterobacteriaceae*, there is a pressing need to find alternative treatment methods. Polymyxin E, commonly known as colistin, has emerged as a critical last-resort agent for treating infections caused by carbapenem-resistant, MDR strains, untreated by other antimicrobials, especially carbapenems [23,24,25,26,27,28]. Colistin, discovered over fifty years ago and commonly known as colistin, is a cationic lipopeptide antibiotic. Unlike carbapenems used in human medicine, colistin is primarily used in animal treatment. In the mid-20th century, colistin gained global, across-the-board use in veterinary medicine. Two commercially available forms of polymyxin E are commonly used: colistin sulphate for oral and topical use (the only approved product in some countries used in pig production to control infections caused by *Enterobacteriaceae*) and colistin methanesulphonate sodium for parenteral use. In addition to its therapeutic purposes, colistin is also commonly used to promote animal growth [29,30]. Currently, the greatest consumption of colistin is associated with poultry treatment practices. The long-lasting misuse and overuse of colistin have been reflected in the dissemination of colistin resistance among microorganisms [24].

Until 2015, all known polymyxin resistance mechanisms were chromosomally mediated [31,32]. The first case of polymyxin resistance, distributed via horizontal gene transfer (HGT), was identified 10 years ago during studies on antimicrobial resistance among bacteria belonging to *Enterobacteriaceae*. The first mobile determinant of polymyxin resistance, the *mcr*-1 gene, was reported in China [31]. The emergence of the mobile colistin resistance (*mcr*) gene was a breakthrough in polymyxin resistance mediated by plasmids. Over the last 10 years, various studies have documented new unknown variants of *mcr* genes ranging from *mcr*-1 to *mcr*-10 [30]. The rapid increase in plasmid-mediated *mcr* gene variants has raised major public health concerns. Occasionally, some *mcr* are noticed on bacterial genomes, indicating a relic of recombination/integration between chromosomes and plasmids [33]. The presence of *mcr* genes can transfer horizontally among microorganisms and further distribute among humans, animals, and the environment [24].

It is particularly dangerous that carbapenem resistance is frequently connected with accompanying resistance to other antimicrobials, including mobile resistance to colistin, representing a pandrug-resistant phenotype [20,34,35]. Coexistence of carbapenem- and colistin resistance determinants is especially worrisome and results in dire public health consequences. Several studies have already reported overlapping carbapenem and colistin resistance in pathogenic strains within the *Enterobacteriaceae* family, such as *Escherichia coli* and *Klebsiella pneumoniae*, from various matrices, especially of clinical origin [36,37,38,39,40]. However, data on the emergence and dissemination of pandrug-resistant bacteria of high clinical significance in the natural environment are severely limited [22].

**Figure 1:**
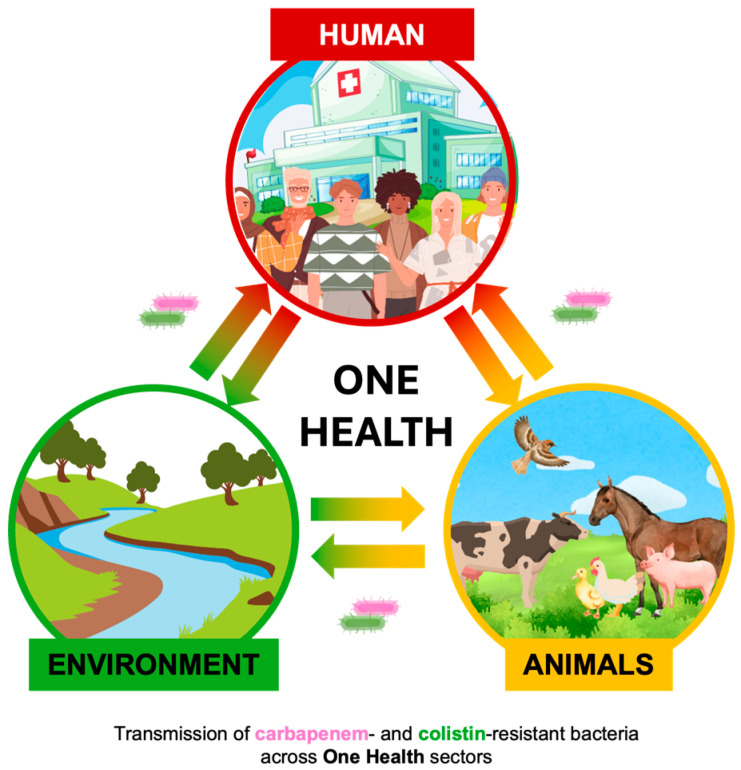
Schematic representation of the dissemination of carbapenem- and colistin-resistant bacteria across the One Health sectors: humans, animals, and the natural environment.

Anthropogenic activities and the associated release of antimicrobials, ARB, and ARGs into the environment have significantly impacted AMR evolution. Aquatic environments, including groundwater and surface water (mainly rivers and lakes), have become a breeding ground for AMR transmission [20,41,42,43,44]. It should be especially emphasized that AMR in the natural environment can provide a reflection of the regional and global clinical resistance situation. Moreover, the differences and similarities in AMR profiles among microorganisms isolated from clinical and natural environments reveal a complex interplay that requires a complex study to fully understand the risk to public health [41,43]. Given the interconnectedness of the natural environment, animals, and humans, the AMR issue requires a One Health approach to evaluate its dissemination and evolution [20] (Figure 1).

Taking the above into account, we focused on the aquatic environment as a particularly important, often overlooked reservoir of resistance to carbapenems and colistin, a major cause of critical infections. The role of the natural water sources as a medium for the spread and evolution of resistance to these critical last-line drugs is crucial for public health protection and has not yet been summarized in the scientific literature. To grasp the core of the problem and facilitate accurate tracking of the spread of AMR to these last-line drugs, our study integrates data from both the scientific literature and whole-genome sequencing. This approach allows for precise monitoring of AMR’s dissemination to carbapenems and colistin, which are reserved for only the most difficult-to-treat diseases. Our review aims to thoroughly examine the role of aquatic environments as reservoirs and hotspots for the spread of resistance to critically important pharmaceuticals, whose retransmission poses a direct threat to the effectiveness of medical treatment. By simultaneously combining bibliographic and genomic data analysis, our study provides a foundation for studying the evolution of AMR in the context of last-line drugs in the natural water environment and tracking its transmission pathways.

## 2. Materials and Methods

### 2.1. Data Sources

In compliance with the Preferred Reporting Items for Systematic reviews and Meta-Analyses (PRISMA) guidelines [45], the articles were selected according to the four criteria: (i) identification, (ii) screening studies, (iii) eligibility, and (iv) inclusion. In this work, two data sources were used: bibliographic and genomic. In the first case, the PubMed and Google Scholar scientific literature databases were surveyed to find reviewed papers published from 1 January to 30 November 2025. In the case of a genomic data source, specific sequences were explored in the Pathogen Detection tool provided by the National Center for Biotechnology Information (NCBI).

### 2.2. Bibliographic Data Search Strategy

The bibliographic data search strategy employed is illustrated in Figure S1 in the Supplementary Materials. A preliminary search of the available scientific literature related to the subject of this review was conducted to identify the keywords to be employed in the advanced search. The keywords used in the search strategy were the following: “((mcr[Title]) OR (colistin[Title]) OR (bla[Title]) OR (carbapenem[Title])) AND ((water[Title]) OR (river[Title]) OR (aquatic[Title]))” in the PubMed database and “TITLE (mcr OR colistin OR polymyxin) AND (bla OR carbapenem) AND (water OR river OR natural OR aquatic)” in the Scopus database. These were tailored to each database. The keywords of all resulting articles are presented in Figure S2. In addition, a reference list of articles was checked manually so as to find adequate scientific publications for this review of data from the literature. After filtering the resulting bibliographic data, 150 scientific publications were selected for this review article and their findings were analyzed.

### 2.3. Genomic Data Search Strategy

Genomes of bacteria isolated from surface waters that possess last-resort antibiotic resistance genes were searched for using the Pathogen Detection tool provided by the National Center for Biotechnology Information (NCBI). To search for genomes with mobile colistin resistance mechanisms, the “AMR genotypes” filter was applied, selecting all variants of the *mcr* genes. This was further refined by overlaying the “Isolation source” filter using the following terms: “river,” “lake,” “ocean,” “sea,” “marine,” “aquatic,” and “water.” Records that did not fit the paper scope, such as those indicating “wastewater,” were manually excluded. To search for genomes carrying carbapenemase genes, we selected carbapenemase genes from “AMR genotypes” filter. Only genes of mechanisms classified as carbapenemases according to the BLDB database were selected [46]. The same “Isolation source” search parameters as above were maintained. For both carbapenemase and colistin resistance genes, only genomes with a complete sequence length were included in the analysis (as indicated by the ‘COMPLETE’ status within the AMR genotype filter). To ensure high data quality, genomes containing only partial ARG sequences or those truncated at the end of a contig were excluded from further investigation. Detailed information regarding the search queries performed in the Pathogen Detection database is provided in Table S1. The complete list of 278 genomes ultimately included in the analysis is available in File S1.

### 2.4. Genome-Based Analyses

The genomic sequences were retrieved from the NCBI database and visualized/analyzed using the Proksee (5-12-2025 version) [47] and CARD Resistance Gene Identifier (RGI 6.0.3, 1.3.1 Tool Version) [48]. Within Proksee, contigs containing the *mcr* and carbapenemase genes were identified. Sequences of the selected contigs were exported from the bacterial genomes using the Geneious Prime software (2025.2.1) and were subsequently analyzed with PlasmidFinder 2.1 [49] to detect plasmid replicon sequences (thresholds of 95% for minimum identity and 60% for minimum % coverage). Contigs harboring successfully identified ARGs and plasmid replicons were further investigated using the COPLA tool [50] to predict their potential host range. Visual comparison of plasmid similarity was performed using Proksee’s built-in BLAST tool (BLAST+ 2.16.0, 1.5.1 Tool Version). Bacterial assignment to specific sequence types (STs) was determined using the MLST 2.0 tool [51]. The similarity of plasmid and whole-genome sequences (Average Nucleotide Identity (ANI) values) was calculated using the FastANI tool (v1.34) [52].

## 3. Results

### 3.1. Carbapenem Resistance in the Natural Aquatic Environment

Antimicrobial resistance to carbapenems is a significant global health issue. More than 80% of ARGs against carbapenems are located on plasmids and can be easily transferred among bacteria via HGT [53]. Carbapenemase-producing microorganisms are frequently isolated not only in hospitals and healthcare facilities but also in the natural environment, such as natural water sources. This raises concerns, as it appears that after the clinical environment, a significant reservoir for carbapenem resistance may exist in the natural aquatic environment. Microorganisms are crucial components of natural aquatic environments, which include groundwater, inland surface waters, seas, and oceans. Freshwater ecosystems include rivers, lakes, streams, ponds, and wetlands, as well as drinking water sources like wells. Marine ecosystems encompass vast oceans, coral reefs, and mangroves [54,55]. All these types of water sources were considered during the data collection process for this review.

A review of bibliographic data on the presence of carbapenem resistance genes in natural aquatic environments is summarized in Table 1. *Escherichia coli* (*n* = 8, 13.5%) and *Klebsiella pneumoniae* (*n* = 10, 16.9%) were identified as the most common carbapenemase-producing isolates. Moreover, carbapenem resistance genes have also been confirmed in several clinically significant bacteria belonging to the *Acinetobacter*, *Enterobacter*, and *Pseudomonas* genera. However, these genes were also found in typical environmental, non-clinical isolates of the genera *Aeromonas*, *Comamonas*, *Shewanella*, *Stenotrophomonas*, and *Vibrio*, indicating that the natural environment is becoming an active reservoir of resistance to last-line drugs. In addition, it should be emphasized that members of the genus *Stenotrophomonas* have inherent resistance to a wide range of β-lactam antibiotics due to two β-lactamases encoded chromosomally (by *bla*_L1_/*bla*_L2_ genes) [56]. Various samples from natural water sources contained such isolates [57,58]. Interestingly, some studies have reported *Stenotrophomonas maltophilia* isolated from seawater that carried the *bla*_OXA-58_ gene [19]. This discovery illustrates that environmental bacteria can acquire and integrate clinically relevant resistance genes, potentially leading to their transmission and contributing to the spread of AMR.

Regardless of taxonomy, various bacteria have been reported to harbor several genetic determinants that enable the production of different carbapenemases. Most of the bibliographic data reports originated from China (17%) and Brazil (11%), while river water samples were the most commonly examined (44%) (Table 1).

**Figure 2:**
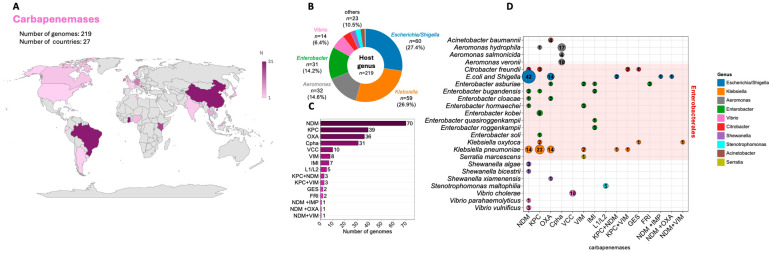
Data on available bacterial genomes detected in natural surface waters carrying carbapenemase genes within their genomes: (**A**)—The geographic distribution of the available genomes; the color intensity of each country is proportional to the number of genomes available from that region. (**B**)—The contribution of individual bacterial genera producing carbapenemases; specific counts are provided for the five most prevalent genera (with the color legend positioned on the right side of the graphic). (**C**)—The contribution of individual carbapenemases whose genes were detected in the genomes of surface water bacteria; notably, the indication of two mechanisms connected by a plus sign (e.g., NDM-1 + KPC-2) denotes the presence of both carbapenemase-encoding genes within the same genome. (**D**)—The carbapenemase–host associations at the genus level, where taxa classified into the order Enterobacterales are highlighted in red. The size of each circle represents the number of genomes found in specific configurations, as indicated by the numerical values within the circles. The specific carbapenemase types classified under the respective sets include NDM-1, NDM-5, NDM-7, and NDM-9; KPC-2 and KPC-3; OXA-23, OXA-48, OXA-65, OXA-69, OXA-181, OXA-204, and OXA-244; and VIM-1, IMP-1, IMI-2, IMI-20, GES-5, FRI-8, FRI-11, VCC-1, L-1, and Cpha.

Based on available genomic data from bacteria isolated from surface waters, we compiled information on carbapenemase-carrying isolates originating from 27 countries worldwide. The largest numbers of genomes were derived from isolates collected in Brazil (*n* = 31; 14.15%), Ghana (*n* = 31; 14.15%), and China (*n* = 22; 10.04%) (Figure 2A). Across all datasets, the predominant hosts of carbapenemase genes were members of the genera *Escherichia*/*Shigella*, *Klebsiella*, and *Enterobacter* (Figure 2B). The most frequently detected carbapenemase families included NDM (*n* = 76, 33.33%), KPC (*n* = 45, 19.73%), and OXA (*n* = 37, 16.22%), and several genomes carried more than one carbapenemase gene (*n* = 9, 4.11%), indicating co-occurrence of multiple resistance determinants within single strains (Figure 2C). Most genomes were classified as *Escherichia coli* (*n* = 60, 27.40%) and *Klebsiella pneumoniae* (*n* = 55, 25.11%), which not only carried the widest diversity of carbapenemase genes but also most commonly accumulated multiple carbapenemase determinants within the same genome (Figure 2D).

Environmental bacteria constitute an important reservoir of natural carbapenem resistance in aquatic settings, mediated by genes such as *bla*_CphA_ (in *Aeromonas* spp.), *bla*_VCC_ (in *Vibrio cholerae*), and *bla*_L_ (in *Stenotrophomonas maltophilia*) (Figure 2D). Clinically relevant carbapenemase genes—including *bla*_NDM_, *bla*_KPC_, and *bla*_OXA_—were also identified in genomes of typically environmental bacteria, such as those belonging to the genera *Vibrio*, *Shewanella*, and *Aeromonas*, underscoring the increasing role of natural aquatic habitats as reservoirs and potential exchange hubs for high-priority resistance genes.

**Figure 3:**
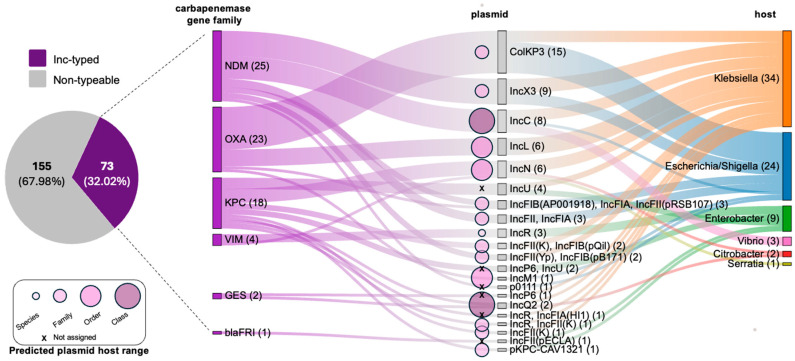
Associations between identified carbapenem resistance genes, specific plasmid replicons, and their bacterial hosts. The pie chart illustrates the proportion of analyzed carbapenemase-encoding genes successfully linked to typed plasmids (Inc-type) versus those where such linkage could not be established (non-typeable). An ARG–plasmid association is defined by the co-localization of an ARG and a specific plasmid replicon sequence on the same genomic contig. The host range of each plasmid, predicted by COPLA, is represented by circle size: species (narrowest), family, order, and class (broadest). Plasmids for which no host range could be confidently determined are marked with an ‘x’ (not assigned). The plasmid–host association indicates the detection of these ARG–plasmid complexes within the analyzed genomes of specific bacterial taxa.

Among the analyzed bacterial genomes, 73 carbapenem resistance genes (32.02%) were successfully linked to specific plasmid replicons based on their co-localization on the same genomic contig (Figure 3). Conversely, for 155 ARGs (67.98%), a definitive plasmid association could not be established. Mobilization was confirmed for six carbapenemase family genes (NDM, OXA, KPC, VIM, GES, and FRI), which were associated with 21 unique plasmid replicon types across six bacterial genera. *Klebsiella* species exhibited the highest diversity in terms of plasmid-mediated resistance, carrying carbapenemase genes on 13 distinct plasmid types. The COPLA tool successfully assigned the analyzed plasmid contigs harboring carbapenemase genes to specific Plasmid Taxonomic Units (PTUs) in 61 out of 73 cases (83.56%), thereby predicting their theoretical host range. Based on the COPLA analysis, the identified carbapenemase-carrying plasmids exhibited a diverse range of potential hosts, spanning from the narrowest at the species level (IncR), through the family level (ColKP3; IncX3; IncFIB(AP001918)_IncFIA_IncFII(pRSB107); IncFII_IncFIA; IncFII(K)_IncFIB(pB171); IncFII(Yp)_ IncFIB(pB171); pKPC-CAV1321; IncR_IncFII(K); IncFII(K)), to the order level (IncN; IncL; IncM1), and reaching the class level (IncC and IncQ2) (Figure 3, File S1). Notably, the IncC plasmid was confirmed as a broad-host-range vector, facilitating the presence of *bla*_NDM_ in both Enterobacterales and *Vibrio* isolates (Figure 3).

**Figure 4:**
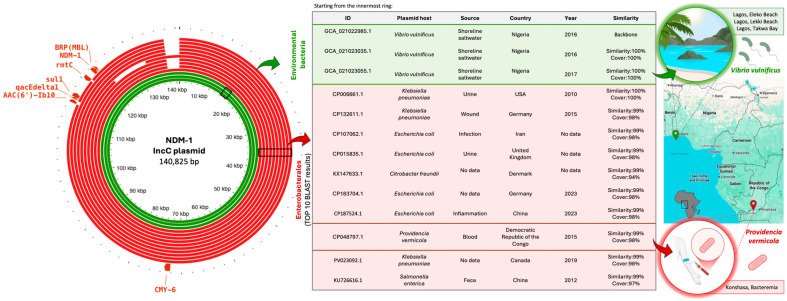
Example of plasmid co-sharing carrying the NDM-1 resistance mechanism gene between environmental bacteria (*Vibrio vulnificus*) and clinically isolated bacteria (Enterobacterales). The left panel presents the IncC plasmid map (140,825 bp). The core plasmid derived from the *Vibrio vulnificus* genome is shown in the innermost ring. Subsequent rings show the BLAST alignment results in green for the environmental bacteria (*V. vulnificus* isolates) and in red for the 10 most similar plasmids retrieved from the NCBI database (hosted by Enterobacterales), which are further characterized in the accompanying table. The right panel visually represents the environments and locations of the plasmid hosts for bacteria isolated from surface waters (top) and for bacteria where a similar plasmid was detected in the closest geographical proximity (bottom).

As the sole non-Enterobacterales representatives within the observed ARG–plasmid–host associations, three *Vibrio* genomes were identified as carriers of the *bla*_NDM-1_ gene on IncC-type plasmids (Figure 3). In-depth analysis of these specific genomes revealed that the plasmids were reconstructed using long-read sequencing technology, which ensured high assembly continuity and provided complete, circularized sequences. Specifically, our comparative plasmid analysis (Figure 4) demonstrated that these *Vibrio vulnificus* isolates, recovered from a coastal beach in Nigeria, carried a plasmid identical/nearly identical to those found in several clinical Enterobacterales isolates, while a related plasmid was also reported in *Providencia vermicola* from the Democratic Republic of Congo. This geographic proximity of environmental and clinical reservoirs, combined with the high mobility of plasmids carrying carbapenemase genes, underscores the potential for inter-species and inter-ecosystem dissemination. Together, these observations illustrate how aquatic environments can act as contact zones where environmental bacteria and clinically derived resistance plasmids co-occur, enabling genetic exchange and contributing to the broader spread of AMR.

### 3.2. Mobile Colistin Resistance in the Natural Aquatic Environment

The global distribution of *mcr* genes, responsible for coding mobile resistance to polymyxin E, is influenced by HGT through plasmids, as well as transposons and integrons [91]. The *mcr* genes are generally harbored by various conjugative and non-conjugative plasmids, but in some isolates, they can also be integrated into the chromosome [92]. Interestingly, it is assumed that because of the variability in the sequence similarity, *mcr* genes have a different genetic origin. The sequence comparison reveals specific variations; the *mcr*-1 gene shares varying degrees of amino acid sequence identity with other *mcr* variants: *mcr*-2 (81%), *mcr*-3 (34%), *mcr*-4 (33%), *mcr*-5 (31%), *mcr*-6 (82%), *mcr*-7 (29%), and *mcr*-8 (31%) (Mondal 2024) [30]. Moreover, various variants of *mcr* genes are possessed by different species of Gram-negative bacteria. In any environment, whether clinical or natural, nearly 90% of all *mcr*-positive isolates are identified as *Escherichia coli* [30,93]. In general, the *mcr*-1 and *mcr*-9 genes are most frequently reported among Enterobacterales, *mcr*-3 in *Aeromonas* sp., and *mcr*-10 in *Enterobacter* sp. [30,94]. The findings from the review of bibliographic data regarding the presence of *mcr* genes in natural aquatic environments are consistent with established knowledge and are summarized in Table 2. *Escherichia coli* was identified as the most common *mcr*-positive isolate (*n* = 13; 46%). However, the presence of *mcr* genes has been confirmed in several clinically important bacterial species, including *Escherichia coli*, *Klebsiella pneumoniae*, and bacteria belonging to *Salmonella* sp. and *Enterobacter* sp. Similarly to the hosts of carbapenem resistance genes (see Table 1), *mcr* genes were also found in bacteria unrelated to clinical settings, originating from typical natural sources such as the genera *Aeromonas*, *Stenotrophomonas*, and *Cupriavidus*. This underscores the significant role of the environment, both as a medium and as a source of typical environmental bacteria that carry resistance to critical drugs. Notably, bacteria from the *Aeromonas* genus have been identified to possess the *mcr*-3 gene in their genomes (*n* = 6). The scientific literature suggests that the origin of *mcr*-3 can be traced back to this Gram-negative bacterium, which is commonly found in soil and water environments. Some studies have shown that *mcr*-3 originated and evolved from *Aeromonas* species, eventually leading to its presence in significant members of the *Enterobacteriaceae* family, such as *Escherichia coli* and *Klebsiella pneumoniae* [95,96,97]. Although we have not found any reports of colistin-resistant representatives of the water environmental genus *Shewanella*, it should be emphasized that the scientific literature indicates this bacterium, found in marine environments and aquatic ecosystems, possesses nonmobile colistin resistance determinants (*nmcr*), which are probably progenitors of the *mcr*-4 gene [33,98].

A review of bibliographic data shows that various microorganisms can simultaneously carry numerous types of *mcr* genes. River water was the most frequently studied sample among natural water reservoirs (*n* = 13). Notably, the majority of these reports originated from China (31%) and Brazil (17%) (Table 2), as in the case of reports regarding carbapenem resistance bacteria isolated from natural water environment (Table 1).

**Figure 5:**
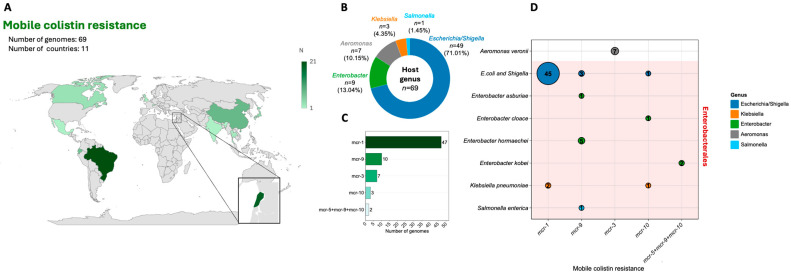
Genomic data from bacteria detected in natural surface waters carrying mobile colistin resistance genes. (**A**)—Geographic distribution of available genomes; the color intensity of each country is proportional to the number of genomes available from that region. (**B**)—Relative abundance of bacterial genera carrying *mcr* genes (color legend shown on the right). (**C**)—Distribution of *mcr* gene variants among genomes from surface-water isolates; the combination of three mechanisms connected with a “+” symbol indicates the simultaneous presence of three *mcr* genes within the same genome. (**D**)—Associations between *mcr* variants and their bacterial hosts at the genus level. Taxa classified within the order Enterobacterales are highlighted in red. The size of each circle represents the number of genomes found in specific configurations, as indicated by the numerical values within the circles.

Based on available genomic data from bacteria isolated from surface waters, we compiled information on *mcr* gene-carrying isolates originating from 11 countries worldwide. The largest numbers of genomes were derived from isolates collected in Brazil (*n* = 21, 30.43%), Lebanon (*n* = 19, 27.54%), and China (*n* = 8, 11.59%) (Figure 5A). The vast majority of reported genomes belonged to members of the genera *Escherichia*/*Shigella* (*n* = 49, 71.01%) (Figure 5B). In total, five unique *mcr* gene variants were detected among bacterial genomes originating from surface waters, with *mcr*-1 being the most prevalent (*n* = 47, 64.38%) (Figure 5C). All *mcr* variants were detected in representatives of Enterobacterales (Figure 5D), with the exception of *mcr*-3, which is strongly associated with the genus *Aeromonas*—a potential progenitor of this resistance mechanism [115].

**Figure 6:**
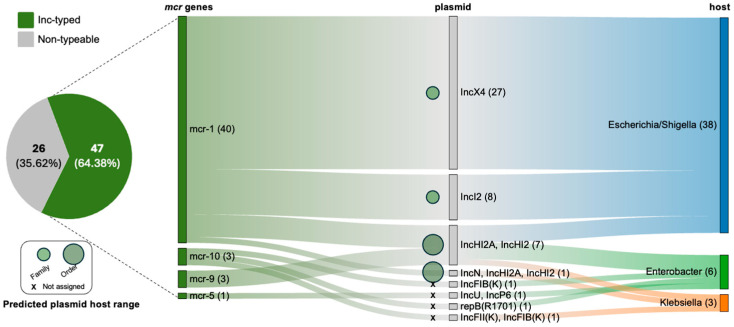
Associations between identified *mcr* genes, specific plasmid replicons, and their bacterial hosts. The pie chart illustrates the proportion of analyzed *mcr* genes successfully linked to typed plasmids (Inc-type) versus those where such linkage could not be established (non-typeable). An ARG–plasmid association is defined by the co-localization of an ARG and a specific plasmid replicon sequence on the same genomic contig. The host range of each plasmid, predicted by COPLA, is represented by circle size: family (narrowest) and order (broadest). Plasmids for which no host range could be confidently determined are marked with an ‘x’ (Not assigned). The plasmid–host association indicates the detection of these ARG–plasmid complexes within the analyzed genomes of specific bacterial taxa.

The mobilization analysis of mobile colistin resistance genes revealed that 47 *mcr* genes (64.38%) were successfully linked to plasmid replicons, while 26 genes (35.62%) remained non-typeable (Figure 6). The *mcr* genes were identified across seven unique plasmid types within the genomes of three distinct bacterial genera belonging to the order Enterobacterales. A high degree of replicon specificity was observed, particularly for *mcr*-1, which was predominantly associated with the IncX4 and IncI2 plasmids in *Escherichia*/*Shigella*. Furthermore, the IncHI2 and IncHI2A plasmids exhibited the greatest diversity, both in terms of the *mcr* variants they carried (*mcr*-1 and *mcr*-9) and their bacterial host range, which included *Escherichia*/*Shigella*, *Enterobacter*, and *Klebsiella*. The COPLA tool successfully assigned the analyzed plasmid contigs harboring *mcr* genes to specific Plasmid Taxonomic Units in 43 out of 46 cases (93.48%), thereby predicting their theoretical host range (File S1). According to the COPLA analysis, these identified *mcr*-carrying plasmids exhibited a diverse range of potential hosts, spanning from the family level (IncX4 and IncI2) to the order level (IncHI2_IncHI2A and IncN_IncHI2_IncHI2A) (Figure 6, File S1).

### 3.3. Co-Resistance to Carbapenems and Colistin Among Bacteria from Environmental Water Sources

Several studies have identified colistin- and carbapenem-resistance genes simultaneously occurring in bacteria isolated from various natural water sources (Table 3). These reports are limited to representatives of Enterobacterales—*Enterobacter* (*n* = 3), *Klebsiella* (*n* = 2), *Escherichia* (*n* = 2), and *Citrobacter* (*n* = 1)—recovered from diverse types of waters across the world, which is consistent with global clinical trends [125]. The authors of these studies highlight direct links between the environmental isolates listed in Table 3 and related clinical isolates [126,127] as well as isolates recovered from discharge water originating from a nearby hospital and a fish farm [120]. Of particular concern is the fact that the waters from which these isolates were obtained included, among others, a lake serving as a source of irrigation water for surrounding villages [128] and coastal waters suitable for primary contact recreation [129].

**Figure 7:**
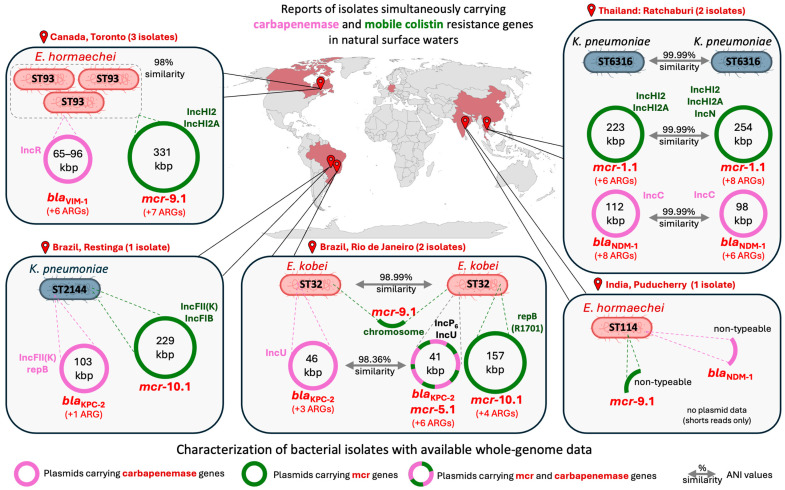
Geographic and genomic characteristics of bacteria isolated from surface waters that simultaneously carried carbapenemase genes and mobile colistin resistance genes. The map shows the locations where such isolates were detected, based on both whole-genome sequencing data and literature reports (data summarized in Table 3). For isolates with available whole-genome data, we describe the host sequence type (ST), as well as the genomic location of carbapenem and/or colistin resistance genes, indicating whether they were plasmid-borne or chromosomally encoded. Cases in which multiple isolates were obtained from the same sample are grouped together, with information on genomic and plasmid variability or similarity (ANI similarity values). When resistance genes were located on plasmids, we report the plasmid incompatibility groups, plasmid sizes, and whether the plasmid carried additional antimicrobial resistance determinants beyond carbapenem and colistin resistance (+n ARGs). For genomes not sequenced with long-read technologies, complete plasmid structures could not be reconstructed. The term ‘non-typeable’ indicates contigs of likely plasmid origin for which a specific incompatibility group or replicon type could not be assigned.

Genomic data derived from available long-read sequencing assemblies highlight the high mobility of carbapenem and colistin resistance determinants within bacterial cells, carried on plasmids together with additional antimicrobial resistance genes (Figure 7). Plasmids encoding resistance to last-resort antibiotics also exhibited substantial variability, despite occurring in bacterial isolates that were otherwise highly similar. Although carbapenemase and *mcr* genes were typically located on separate plasmids, co-localization on a single plasmid was also observed, for instance, in *Enterobacter kobei.*

Although co-resistant bacteria pose a significant threat, they currently appear to be mostly sporadic; nevertheless, it remains crucial to implement appropriate measures to limit their dissemination [125]. The reported findings also indicate that the detection of multiple isolates (2–10) within a single sample is unlikely to be accidental, suggesting either in situ proliferation or a strong point-source contamination event.

## 4. Discussion

The findings of this review highlight an important aspect of the ‘One Health’ approach, which encompasses human, animal, and environmental health sectors—the last of which, unfortunately, is often overlooked in global surveillance. Our work is intended to initiate intensified efforts to ensure that carbapenems and colistin remain effective treatment options in clinical settings. However, the presented findings extend beyond expected patterns, showing that the aquatic environment serves as a dynamic evolutionary interface. The results of this review are consistent with global monitoring trends, where the high prevalence of ARGs in Enterobacterales is a logical consequence of their WHO priority status and focused surveillance. However, the path to a comprehensive global strategy for monitoring and combating AMR faces certain challenges. Although this review synthesizes data from both bibliographic sources and genomic analyses—covering 41 countries across several continents—significant geographical gaps remain. There is a notable disproportion in the number of available reports, with a dominance of data originating from China and Brazil. Consequently, many regions remain underrepresented, highlighting the urgent need for more geographically diverse surveillance to achieve a truly global understanding of the mechanisms driving the dissemination of carbapenem and colistin resistance.

**Figure 8:**
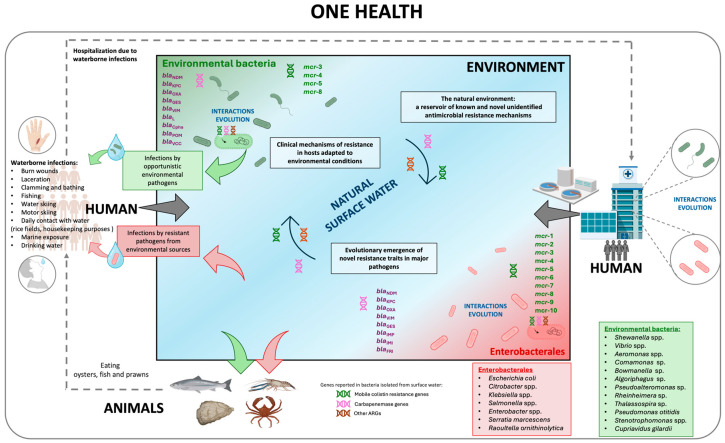
Schematic summary of data regarding the prevalence of bacteria carrying carbapenem resistance genes and mobile colistin resistance genes in natural surface waters. Based on the data collected in this study, the figure highlights the fraction of last-resort antibiotic resistance genes detected in waterborne Enterobacterales, as well as in environmental bacteria identified in the review. The specific host taxa for these resistance mechanisms are listed in the bottom right corner. Potential transmission routes of the aforementioned bacterial taxa are presented, considering the water environment-to-human pathway based on [136,137,138], the environment-to-animal pathway [136,139,140,141,142,143,144], and the animal-to-human pathway [136,140], within the context of the One Health concept. Green arrows indicate potential transmission routes for bacteria classified as ‘environmental bacteria’, while red arrows represent those classified as ‘Enterobacterales’. Gray arrows denote the potential influx of bacteria into the ‘Environment’ component. Dashed lines indicate transmission occurring independently of the ‘Environment’ component.

The data presented in the preceding sections show that genetic determinants of carbapenem and colistin resistance are widespread among bacteria inhabiting aquatic environments—both within the native environmental microbiota and among members of Enterobacterales. These two distinct microbial groups coexist and interact within water systems, creating opportunities for evolutionary adaptation, HGT, and ecological expansion. Such continuous circulation of microorganisms and their resistance determinants across natural environments, animals, and humans reflects the interconnected dynamics central to the One Health framework (Figure 8).

An intriguing observation emerges regarding the role of *Escherichia coli* in aquatic resistomes. Although *E. coli* was identified as the predominant host for *mcr* and carbapenemase genes in surface waters, its associated plasmids—typically IncX3, ColKP3, and IncF for carbapenemases and IncX4 or IncI2 for *mcr*—appear to exhibit a more restricted host range, potentially limiting their dissemination to indigenous aquatic genera such as *Vibrio*, *Aeromonas*, and *Pseudomonas* [132,133]. In contrast, our findings suggest that other *Enterobacterales* species, particularly *Klebsiella* and *Enterobacter*, may play a more pivotal role as high-risk reservoirs. Specifically, *Klebsiella* is recognized as a critical hub for the accumulation and transmission of resistance determinants [42,134], while together with *Enterobacter*, it serves as a primary driver for the evolution of AMR across diverse environments [135]. These taxa were associated with broad-host-range scaffolds, including IncC and IncQ2, which facilitate cross-taxonomic resistance transfer. While the current scarcity of environmental genomic data warrants caution, these patterns suggest that the ecological risk of AMR dissemination in water bodies is driven by the plasticity of the plasmid scaffold and the fitness of the intermediary host.

Wastewater discharges, runoff, and other human-associated inputs represent a major pathway through which clinically associated bacteria, including healthcare-linked pathogens, are released into surface waters [145]. In this way, aquatic ecosystems become recipients of genetic material previously confined to clinical settings, including last-resort resistance determinants located on mobile genetic elements. Our review indicates that plasmids such as IncQ2 and IncC serve as particularly critical vectors for transmission beyond the Enterobacterales order due to their broad predicted host range. By enriching the environmental resistome with such clinically relevant components, these inputs create conditions that promote extensive interactions between hospital-derived strains and the complex native microbiota. Within this shared habitat, the boundaries between environmental and clinical microbial communities begin to blur. Close coexistence facilitates HGT, enabling the transfer of antimicrobial resistance genes across taxonomic and ecological barriers. Consequently, microorganisms that are already well adapted to natural conditions may acquire highly efficient resistance systems characteristic of clinical pathogens, potentially increasing their ecological competitiveness.

Importantly, this exchange may be bidirectional. Numerous studies indicate that several clinically relevant AMR mechanisms, including resistance to colistin and carbapenems, have environmental origins [89,97,146,147]. Thus, it is plausible to consider the aquatic environment as a cryptic reservoir where yet-undiscovered resistance mechanisms may reside, diversify, and potentially become mobilized within natural habitats [148]. Currently, environmental bacteria are well known to possess intrinsic, chromosomally encoded carbapenem resistance genes—including *bla*_L_ (*Stenotrophomonas maltophilia*), *bla*_CphA_ (*Aeromonas* spp.), *bla*_POM_ (*Pseudomonas otitidis*), and *bla*_VCC_ (*Vibrio cholerae*). While our analysis failed to demonstrate the mobility of these determinants (specifically their plasmid-borne localization), the emergence of their mobile variants in clinical settings has already been documented [149]. Such novel or currently overlooked variants may ultimately enter clinical settings, where continuous and intense selective pressures facilitate their emergence as significant threats [147].

Numerous reports describe infections caused by environmental bacteria which, as shown in our review, frequently harbor genes conferring resistance to last-resort antibiotics. These infections include open-wound and burn-related injuries following contact with water and have been attributed to diverse taxa such as *Vibrio* spp., *Shewanella* spp., *Aeromonas* spp., *Escherichia coli*, *Enterobacter* spp., *Klebsiella pneumoniae*, *Serratia marcescens*, and *Citrobacter* spp. [137,138,140,150]. In several cases, infections resulted in severe clinical outcomes, including limb amputation or death. Transmission may occur during routine occupational activities [140,150], through recreational exposure [140], or through the use of river water as a drinking source [136]. Animals also constitute an important vector: fish, oysters, and shrimp can both carry and become infected with opportunistic environmental bacteria [136,139,140,141], as well as Enterobacterales resistant to carbapenems or/and colistin [141,142,143].

Hospitalization due to waterborne infections commonly requires antimicrobial treatment; however, increasing resistance to first-line antibiotics significantly constrains therapeutic options [138,151]. As a result, last-resort antibiotics are increasingly used, including in severe or rapidly progressing infections among immunocompetent individuals [152]. Continued acquisition of last-resort antibiotic resistance among these taxa could therefore further complicate the management of water-associated infections.

The evidence presented above indicates that such processes are already underway and are likely to intensify in the future, partly as a consequence of climate change [153]. We may increasingly observe both the emergence of environmental bacteria in clinical settings and the re-entry of hospital-associated pathogens into the clinic via waterborne infections. This scenario provides environmental bacteria with expanding opportunities to enter clinical ecosystems, where frequent contact with clinically relevant pathogens and strong selective pressures facilitate further acquisition of antimicrobial resistance mechanisms. Ultimately, these bacteria may again be released from healthcare facilities into surface waters through wastewater effluents, thereby closing the cycle of bacterial circulation across the interconnected components of the One Health framework.

Taken together, these findings underscore that aquatic environments function not merely as passive recipients, but as dynamic, evolutionarily active hubs that mediate the continuous exchange, adaptation, and dissemination of high-risk antimicrobial resistance determinants.

When investigating carbapenem and colistin resistance in aquatic environments, it is essential to recognize that many environmental bacteria possess intrinsic, taxon-specific resistance mechanisms that may resemble acquired resistance phenotypes. This is particularly relevant when screening for clinically significant determinants such as the “Big Five” carbapenemases (KPC, NDM, VIM, IMP, and OXA-48-like), whose detection in environmental isolates can be confounded by naturally occurring β-lactamase activities. Therefore, robust risk assessment requires confirmation of phenotypic findings using molecular approaches capable of distinguishing intrinsic resistance from horizontally acquired ARGs. Whole-genome sequencing, especially long-read technologies, provides a powerful framework for this purpose, as it enables reconstruction of complete plasmid sequences and resolves the genomic context of resistance genes with high precision. Such data are invaluable for tracking the mobility, dissemination pathways, and evolutionary dynamics of clinically relevant ARGs in natural settings. Taxonomic identification remains a critical first step, as knowledge of species-specific intrinsic resistomes, ecological niches, and pathogenic potential allows researchers to interpret resistance profiles more reliably and to differentiate environmental background signals from emerging public health threats. However, investigating native aquatic bacteria remains paramount, regardless of their intrinsic resistance. Unlike transient clinical strains, these indigenous species are highly fitness-adapted to natural waters, allowing them to effectively stabilize and maintain acquired ARGs like *bla*_NDM_ or *bla*_KPC_. By providing a stable genomic backbone for clinical determinants, these well-adapted populations turn into permanent environmental reservoirs, ensuring the long-term persistence of last-resort resistance in the ecosystem.

## 5. Conclusions and Future Directions

### 5.1. Public Health and One Health Implications

Our review identifies two distinct facets of carbapenem and colistin resistance within natural aquatic environments. The first involves allochthonous Enterobacterales, which act as the primary vectors for clinically significant ARGs, frequently localized on mobile plasmids. The second facet comprises the autochthonous aquatic microbiota, which typically harbor intrinsic, non-mobile resistance mechanisms. Natural water bodies serve as a dynamic interface for interaction between these two bacterial fractions.

A particularly critical finding is the simultaneous presence of both carbapenem and colistin resistance determinants within single bacterial cells, even co-located on the same plasmid. This phenomenon of co-resistance on shared MGEs highlights the extreme risk of multidrug resistance dissemination in the aquatic environment.

The findings of this review demonstrate that natural aquatic environments have transitioned from passive recipients of pollution to active, stable reservoirs for carbapenem and colistin resistance. This evolution poses a direct threat to public health by creating environmental routes for high-risk pathogens that can bypass traditional clinical surveillance. We conclude that the presence of genes that provide resistance to last-resort options in both Enterobacterales and autochthonous microbiota increases the risk of community-acquired infections that are inherently difficult to treat. Ultimately, gaining a deeper understanding of these environmental reservoirs is essential to safeguard the clinical efficacy of carbapenems and colistin, ensuring they remain viable treatment options for life-threatening infections.

To effectively address the global AMR crisis, the environmental sector should be recognized as an important driver of resistance. Aquatic systems, in particular, act as a primary reservoir where plasmids emerge as significant vectors for carbapenemase and *mcr* gene dispersal. From a One Health perspective, these water bodies represent the missing link; ignoring them compromises the efficacy of interventions in other sectors by allowing resistance to circulate freely between human, animal, and environmental compartments.

### 5.2. Limitations and Future Research

The data synthesized in this review highlight three primary strategic directions for future research concerning the prevalence and dynamics of critical resistance genes in aquatic environments:

- Monitoring allochthonous vectors: Research should continue to focus on the detection of Enterobacterales carrying ARGs against last-resort antibiotics. As the primary “introducers” of clinically significant, mobile ARGs into natural waters, these organisms represent the most direct threat to public health. While this area is currently the most extensively studied, continued surveillance is essential to track emerging high-risk clones.
- Investigating native recipients: There is an urgent need to shift focus toward identifying clinically relevant ARGs within the natural (autochthonous) microbiota. This will allow for an assessment of which environmental organisms serve as the most effective “recipients” of critical ARGs and which MGEs play the most significant roles in the stabilization and long-term environmental persistence of AMR. Based on our findings, particular attention should be directed toward the genera *Aeromonas*, *Pseudomonas*, *Vibrio*, *Shewanella*, and *Comamonas*. This area remains significantly under-researched, with genomic data for these groups still being relatively scarce.
- Evolution of intrinsic resistomes: It is crucial to monitor known intrinsic resistance mechanisms and discover novel environmental determinants. Tracking the specific genes—both in the environment and in clinical settings—will enable researchers to observe the potential transition of innate ARGs toward mobility, providing an early warning system for future clinical threats.

Furthermore, a significant limitation of currently available data is the high degree of genomic fragmentation, which obscures critical ARG–plasmid associations and limits comparative analyses. The adoption of long-read sequencing technologies is essential to reconstruct complete plasmid sequences. Such high-resolution data will enable precise tracking of the dissemination and structural evolution of last-resort resistance across all One Health sectors.

## Figures and Tables

**Table 1:** Summary of a review of bibliographic data on the occurrence of carbapenem resistance genes and their hosts in samples from natural aquatic environments.

Carbapenem Resistance Gene	Bacterial Host	Environmental Water Type	Country	Reference
-	*Aeromonas veronii*, *Pseudomonas aeruginosa*, *Stenotrophomonas* spp.	natural (forest) and bore hole water sources	Algeria	[59]
*bla*_OXA-48-like_ (*bla*_OXA-538_)	*Shewanella xiamenensis*	river water		[60]
*bla*_GES-5_	*Comamonas* spp.	lake water, wetland	Australia	[61]
-	*Klebsiella pneumoniae*	river water	Bangladesh	[62]
*bla*_GES_, *bla*_KPC_, *bla*_NDM_, *bla*_OXA-51_	*Enterobacter cloacae*, *Aeromonas caviae*, *Acinetobacter venetianus*	river water	Brazil	[63]
*bla*_KPC-2_	*Enterobacter kobei*	coastal water		[64]
*bla*_KPC-2_	*Enterobacter cloacae*	coastal water		[65]
*bla*_NDM-1_	*Klebsiella pneumoniae*	coastal water		[66]
*bla*_NDM-1_	*Raoultella ornithinolytica*	well water	China	[67]
*bla*_NDM-1,_ *bla*_KPC-2_	*Citrobacter* spp.	river water, pond water		[68]
*bla*_KPC-2_	*Raoultella ornithinolytica*	well water		[69]
*bla*_NDM-1_	-	drinking water supply system		[70]
*bla*_OXA-58_	*Pseudomonas* sp., *Rheinheimera* sp., *Stenotrophomonas* sp., *Shewanella* sp., *Raoultella* sp., *Vibrio* sp., *Pseudoalteromonas* sp., *Algoriphagus* sp., *Bowmanella* sp., *Thalassospira* sp.	seawater		[19]
*bla*_OXA-69_	*Acinetobacter baumannii*	river water		[71]
*bla*_NDM-5_	*Escherichia coli*	river water	France	[72]
-	*Escherichia coli*	river water		[73]
*bla_VCC_*_-1_	*Vibrio cholerae*	coastal waters	Germany	[74]
*bla*_POM-1_	*Pseudomonas otitidis*	river water	Ghana	[75]
*bla*_OXA-181_, *bla*_NDM-5_, *bla*_OXA-48_	*Escherichia coli*, *Klebsiella pneumoniae*, *Enterobacter cloacae*	river water, pond water		[76]
*bla*_NDM-1,_ *bla*_NDM-2_, *bla*_NDM-5_	*Serratia marcescens*, *Escherichia coli*, *Klebsiella pneumoniae*	lake water, pond water	India	[77]
*bla*_NDM_, *bla*_OXA-48_	*Klebsiella pneumoniae*	river water		[78]
*bla*_NDM_, *bla*_OXA-48-like_, *bla*_KPC_	*Klebsiella pneumoniae*	river water		[79]
*bla*_OXA-48_, *bla*_KPC_, *bla*_NDM-5_	*Klebsiella pneumoniae*, *Escherichia coli*	seawater	Ireland	[80]
*bla*_IMP_	*Escherichia coli*	river water	Japan	[81]
*bla*_OXA-731_	*Shewanella* sp.	drinking water storage system	Myanmar	[82]
*bla*_IMP_, *bla*_VIM_, *bla*_OXA-51_, *bla*_OXA-58_, *bla*_IMP-1_, *bla*_VIM-2_	*Acinetobacter* spp.	river water	Poland	[83]
-	*Acinetobacter* spp.	river water		[84]
*bla*_KPC-3_	*Klebsiella pneumoniae*	river sediment	Portugal	[85]
*bla*_GES-5_, *bla*_KPC-3_, *bla*_NDM-1_	*Klebsiella pneumoniae*, *Enterobacter* spp., *Citrobacter* spp.	river water		[86]
*bla*_CphA_, *bla*_L1_, *bla*_OXA-48-like_, *bla*_VIM-2_	*Aeromonas* spp., *Stenotrophomonas maltophilia*, *Stenotrophomonas xiamenensis*, *Pseudomonas* spp.	river water		[57]
*bla*_NDM-5_	*Escherichia coli* ST410	reservoir water	Singapore	[87]
-	*Escherichia coli*, *Klebsiella pneumoniae*	river water	Tanzania	[88]
*bla*_OXA-204_, *bla*_NDM-1_	*Shewanella* spp.	river water	Tunisia	[89]
*bla*_IMI-2_, *bla*_L1_	*Enterobacter asburiae*, *Stenotrophomonas maltophilia*	lake water, pond water	USA	[58]
*bla*_OXA-252_, *bla*_OXA-547_	*Shewanella xiamenensis*	water canals	Vietnam	[90]

**Table 2:** Summary of a review of bibliographic data on the presence of *mcr* genes and their hosts in samples from natural aquatic environments.

Mobile Colistin Resistance Gene	Bacterial Host	Environmental Water Type	Country	Reference
*mcr*-5.1	*Cupriavidus gilardii*	well water	Algeria	[99]
*mcr*-1.1	*Escherichia coli*	drinking water	Armenia	[100]
*mcr*-1	*Escherichia coli*	surface water	Bangladesh	[101]
*mcr*-3	*Escherichia coli*	pond water		[102]
*mcr*-1, *mcr*-1.26	-	river water	Brazil	[103]
*mcr*-3, *mcr*-7.1	-	water from a recreation club		[104]
*mcr*-1, *mcr*-2, *mcr*-3, *mcr*-4, *mcr*-5, *mcr*-6, *mcr*-7, *mcr*-8, *mcr*-9	*Acinetobacter* spp., *Enterobacter* spp.	urban recreational estuary water		[105]
*mcr*-1	*Escherichia coli* ST683/CC155	touristic coastal water		[106]
*mcr*-3, *mcr*-3.6	*Aeromonas veronii* TR112	river water		[107]
*mcr*-1	-	river water	China	[108]
*mcr*-1	*Escherichia coli*	river water		[109]
*mcr*-1	*Escherichia coli*	watershed		[110]
*mcr*-1, *mcr*-3	*Escherichia coli*, *Enterobacter cloacae*, *Aeromonas veronii*, *Aeromonas hydrophila*	river water		[111]
*mcr*-1	-	drinking water supply system		[70]
*mcr*-3	*Aeromonas* spp.	river water		[112]
*mcr*-1	*Escherichia coli*	well water		[69]
*mcr*-3	*Stenotrophomonas maltophilia*	lake water		[113]
*mcr*-1	*Escherichia coli*	river water		[114]
*mcr*-3	*Aeromonas* spp.	river water, well water, mud water, surface water	Ghana	[115]
*mcr*-1, *mcr*-2, *mcr*-3	*Salmonella* spp.	river water, lake water	India	[116]
*mcr*-1	*Escherichia coli*	irrigation water	Lebanon	[117]
*mcr*-3, *mcr*-5	-	river water storm water	South Africa (Western Cape)	[92]
*mcr*-10	*Enterobacter cloacae*	river water	Switzerland	[118]
*mcr*-1	*Escherichia coli*	river water		[119]
*mcr*-1, *mcr*-8, *mcr*-9	*Escherichia coli*	surface water	Thailand	[120]
*mcr*-3, *mcr*-4, *mcr*-5, *mcr*-8	*Aeromonas veronii* *Aeromonas media*	springs, ponds, drinking water	Turkey	[121]
*mcr*-3	*Aeromonas jandaei*	surface water	USA	[122]
*mcr*-1.1	*Escherichia coli*	irrigation water		[123]
*mcr*-1	-	lake water, river water, groundwater	Vietnam	[124]

**Table 3:** Summary of information on isolates simultaneously carrying genetic determinants for carbapenem resistance (CRG column) and mobile colistin resistance (MCR column). The data is derived from both a comprehensive literature search and the analysis of publicly available genomes in the NCBI database. Isolates for which full genomic data is available (indicated by the Assembly ID) are further characterized in detail in Figure 7.

Bacterial Host	Isolation Source	Year	Location	CRGs	MCR	No. of Isolates	Detection Method	Assembly ID	Reference
*Klebsiella* *pneumoniae*	Surface water	2021	Brazil: Restinga	*bla*_KPC-2_	*mcr*-10.1	1	WGS	GCA_052220985.1	-
*Enterobacter* *hormaechei*	Surface water	2015	Canada: Ontario	*bla*_VIM-1_	*mcr*-9.1	3	WGS	GCA_015910325.1 GCA_015910345.1 GCA_022023895.1	[126]
*Klebsiella* *pneumoniae*	Natural water sources	2022	Thailand: Ratchaburi	*bla*_NDM-1_	*mcr*-1.1	2	WGS	GCA_029542165.1 GCA_029542185.1	[127]
*Enterobacter* *hormaechei*	Eutrophic lake	2021	India: Puducherry	*bla*_NDM-1_	*mcr*-9.1	1	WGS	GCA_025290855.1	[128]
*Enterobacter kobei*	Coastal Water	2014	Brazil: Rio de Janeiro	*bla*_KPC-2_	*mcr*-9.1 *mcr*-5.1 *mcr*-10.1	1	WGS	GCA_024623675.1	[129]
		2013			*mcr*-9.1	1		GCA_024623685.1	
*Escherichia coli*	River	2021	China: Shandong Province	*bla*_NDM_	*mcr*-1	10	WGS	-	[130]
*Escherichia coli*	River	2021	Thailand: Nongkhai Province	*bla*_OXA-48_	*mcr*-8	2	PCR	-	[120]
					*mcr*-9.1	2			
					*mcr*-8 *mcr*-9	6			
*Citrobacter freundii*	River	2022	Germany: Lower Saxony	*bla*_KPC-2_ *bla*_VIM-1_	*mcr*-9	1	WGS	-	[131]

CRGs—carbapenem resistance genes; MCR—mobile colistin resistance.

## Data Availability

No new data were created or analyzed in this study. Data sharing is not applicable to this article.

## Supplementary Materials

The following supporting information can be downloaded at https://www.mdpi.com/article/10.3390/antibiotics15020113/s1. Figure S1: PRISMA flowchart showing the results of the literature search and the screening process for this review. Figure S2: A keyword co-occurrence map, considering papers containing keywords used in the bibliographic data search strategy. Circle size is proportional to the number of co-occurrences of a particular keyword and clustering by color is based on article publication date. The map was created with the use of VOSviewer (v1.6.16; 2020, Centre for Science and Technology Studies, Leiden University, the Netherlands). Table S1: NCBI Pathogen Detection query details. File S1: Genomic datasets used in this study.
