# Supplementary material for: Silent Threat Evolution: Critically Important Carbapenem and Colistin Resistance Genes in the Natural Aquatic Environment
Source: Antibiotics (Basel). 2026 Jan 23;15(2):113. doi: 10.3390/antibiotics15020113 (PMC12937479; doi:10.3390/antibiotics15020113)
Supplement: Supplementary file 1 [file antibiotics-15-00113-s001.zip › Supplementary materials.pdf]

antibiotics

Supplementary materials

Silent Threat Evolution: Critically Important Carbapenem and Colistin Resistance Genes in the Natural Aquatic Environment

Małgorzata Czatzkowska [1,†] and Damian Rolbiecki [2,*,†]

[1] Department of Water Protection Engineering and Environmental Microbiology, Faculty of Geoengineering, University of Warmia and Mazury in Olsztyn, 10-720 Olsztyn, Poland; malgorzata.czatzkowska@uwm.edu.pl
[2] European Regional Centre for Ecohydrology of the Polish Academy of Sciences, 90-364 Lodz, Poland
* Correspondence: d.rolbiecki@erce.unesco.lodz.pl
† These authors contributed equally to this work.

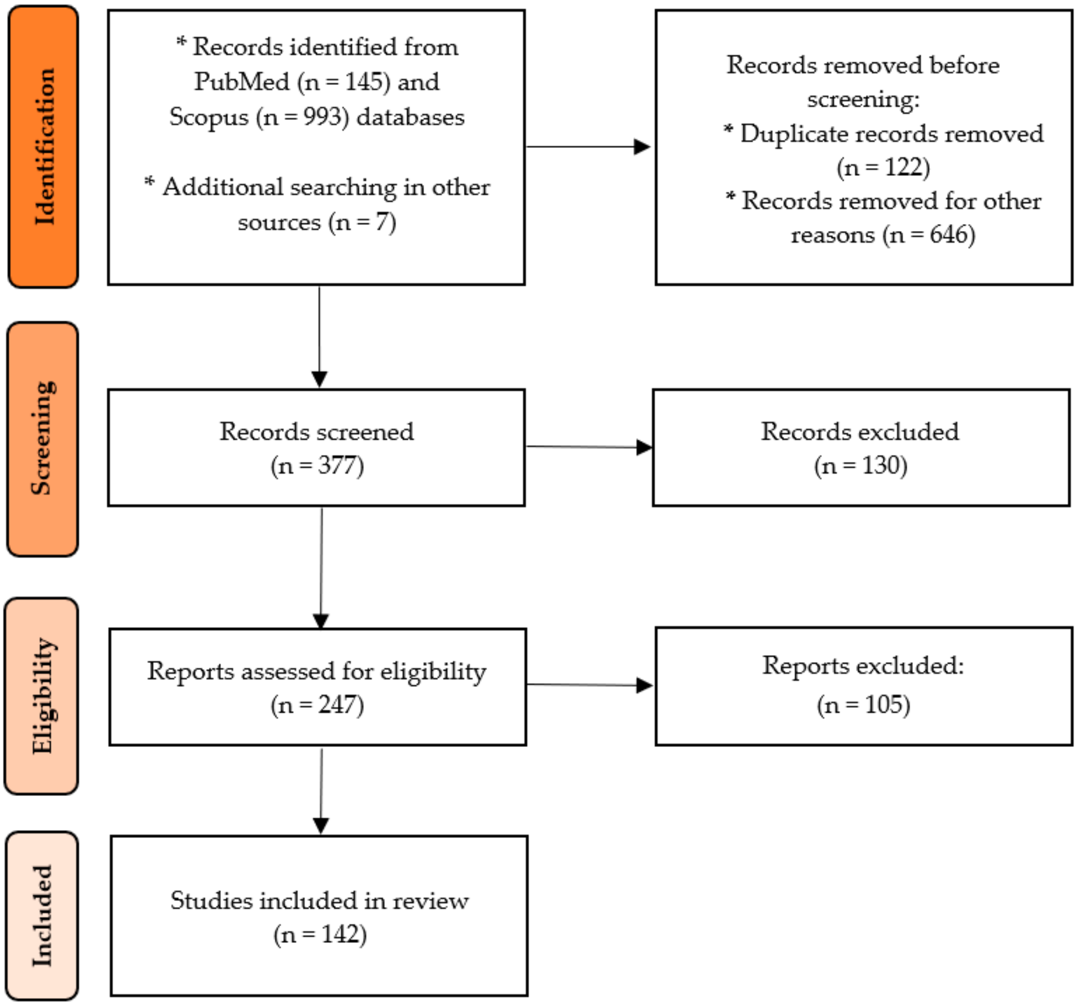

Figure S1. PRISMA flowchart showing the results of the literature search and the screening process for this review.

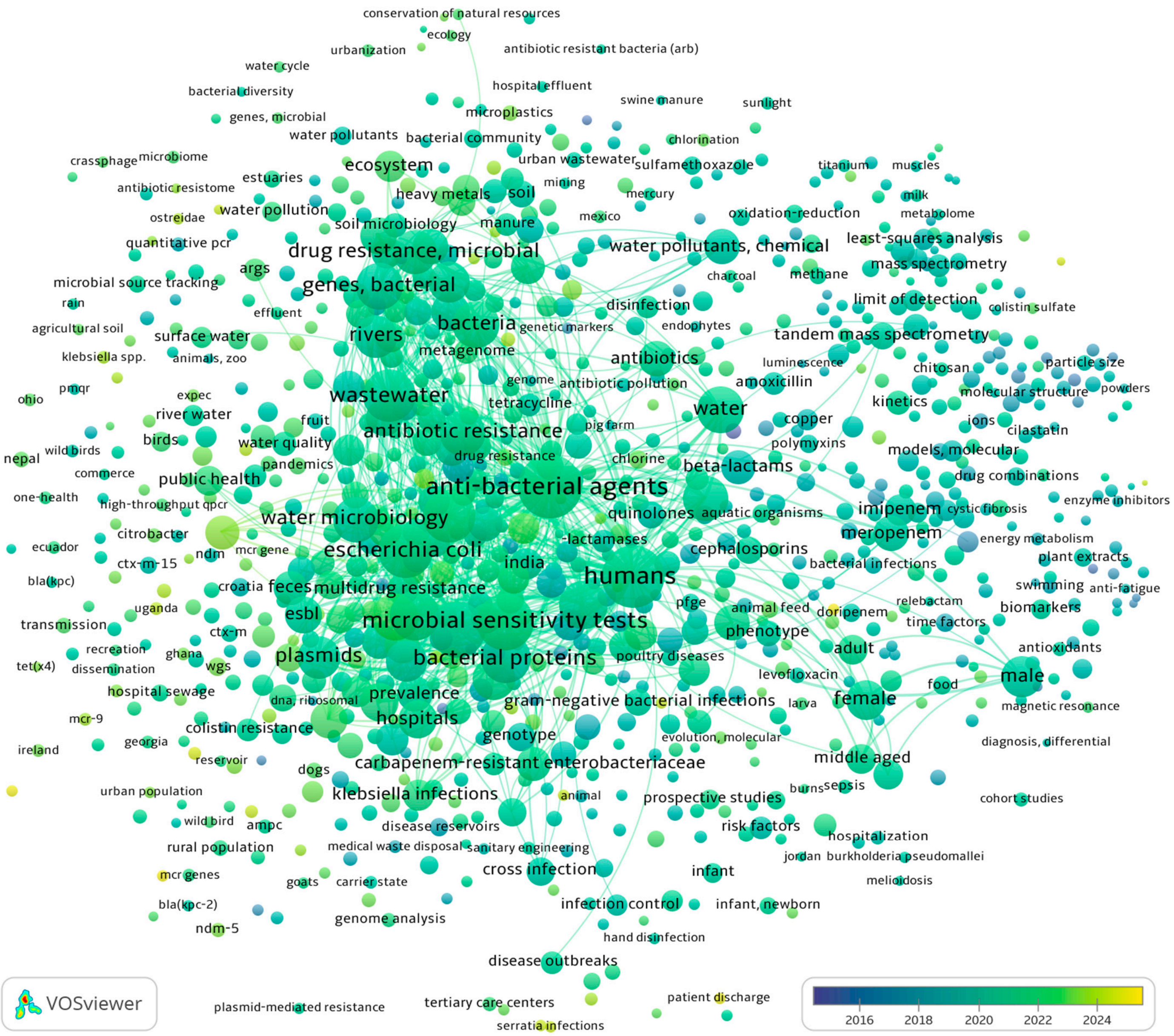

Figure S2. A keyword co-occurrence map, considering papers containing keywords used in the bibliographic data search strategy. Circle size is proportional to the number of co-occurrences of a particular keyword and clustering by color is based on article publication date. The map was created with the use of VOSviewer (v1.6.16; 2020, Centre for Science and Technology Studies, Leiden University, The Netherlands).

Table S1. NCBI Pathogen Detection query details

Target	**NCBI Pathogen Detection query details**
mcr	https://www.ncbi.nlm.nih.gov/pathogens/isolates/#AMR_genotypes:(%22mcr-1.1=COMPLETE%22%20%22mcr-9.1=COMPLETE%22%20%22mcr-9.2=COMPLETE%22%20%22mcr-10.1=COMPLETE%22%20%22mcr-9=COMPLETE%22%20%22mcr-3.1=COMPLETE%22%20%22mcr-3=COMPLETE%22%20%22mcr-1=COMPLETE%22%20%22mcr-3.5=COMPLETE%22%20%22mcr-1.26=COMPLETE%22%20%22mcr-8.1=COMPLETE%22%20%22mcr-5.1=COMPLETE%22%20%22mcr-4.3=COMPLETE%22%20%22mcr-8.2=COMPLETE%22%20%22mcr-10=COMPLETE%22%20%22mcr-1.32=COMPLETE%22%20%22mcr-3.2=COMPLETE%22%20%22mcr-3.39=COMPLETE%22%20%22mcr-2.1=COMPLETE%22%20%22mcr-

	1.2=COMPLETE%22%20%22mcr-4.2=COMPLETE%22%20%22mcr-3.4=COMPLETE%22%20%22mcr-4.6=COMPLETE%22%20%22mcr-1.5=COMPLETE%22%20%22mcr-8=COMPLETE%22%20%22mcr-3.19=COMPLETE%22%20%22mcr-3.17=COMPLETE%22%20%22mcr-3.20=COMPLETE%22%20%22mcr-2.3=COMPLETE%22%20%22mcr-3.21=COMPLETE%22%20%22mcr-3.3=COMPLETE%22%20%22mcr-1.7=COMPLETE%22%20%22mcr-5=COMPLETE%22%20%22mcr-3.40=COMPLETE%22%20%22mcr-3.24=COMPLETE%22%20%22mcr-1.18=COMPLETE%22%20%22mcr-3.29=COMPLETE%22%20%22mcr-1.27=COMPLETE%22%20%22mcr-3.11=COMPLETE%22%20%22mcr-3.16=COMPLETE%22%20%22mcr-4=COMPLETE%22%20%22mcr-11.1=COMPLETE%22%20%22mcr-1.12=COMPLETE%22%20%22mcr-3.6=COMPLETE%22%20%22mcr-3.22=COMPLETE%22%20%22mcr-4.9=COMPLETE%22)%20AND%20isolation_source:(%22river%20water%22%20%22jin%20river%20water%20w2%22%20%22jin%20river%20water%20w5%22%20%22river%22%20%22river%20samples%22%20%22river%20samples1%22%20%22river%20samples2%22%20%22river%20samples3%22%20%22river%20water1%22%20%22eutrophic%20lake%22%20%22seawater%22%20%22sea%20water%22%20%22estuarine%22%20%22surface%20water%22%20%22freshwater%22%20%22coastal%20water%22%20%22freshwater%20sample%20from%20downstream%20of%20wastewater%20treatment%20plant%22%20%22natural%20water%20sources%20close%20by%20farm%20or%20community%22)
NDM	https://www.ncbi.nlm.nih.gov/pathogens/isolates/#AMR_genotypes:(%22blaNDM-1=COMPLETE%22%20%22blaNDM-5=COMPLETE%22%20%22blaNDM-7=COMPLETE%22%20%22blaNDM-4=COMPLETE%22%20%22blaNDM=COMPLETE%22%20%22blaNDM-9=COMPLETE%22%20%22blaNDM-14=COMPLETE%22%20%22blaNDM-6=COMPLETE%22%20%22blaNDM-19=COMPLETE%22%20%22blaNDM-2=COMPLETE%22%20%22blaNDM-13=COMPLETE%22%20%22blaNDM-50=COMPLETE%22%20%22blaNDM-24=COMPLETE%22%20%22blaNDM-16b=COMPLETE%22%20%22blaNDM-66=COMPLETE%22%20%22blaNDM-23=COMPLETE%22%20%22blaNDM-3=COMPLETE%22)%20AND%20isolation_source:(%22river%20water%22%20%22river%22%20%22riverwater%22%20%22river%20estuary%22%20%22river%20upstream%201%22%20%22eutrophic%20lake%22%20%22sea%22%20%22seawater%22%20%22surface%20water%22%20%22shoreline%20saltwater%22%20%22natural%20water%20sources%20close%20by%20farm%20or%20community%22%20%22stream%20water%22%20%22estuary%22)
KPC	https://www.ncbi.nlm.nih.gov/pathogens/isolates/#AMR_genotypes:(%22blaKPC-2=COMPLETE%22%20%22blaKPC-3=COMPLETE%22%20%22blaKPC-4=COMPLETE%22%20%22blaKPC=COMPLETE%22%20%22blaKPC-33=COMPLETE%22%20%22blaKPC-31=COMPLETE%22%20%22blaKPC-6=COMPLETE%22%20%22blaKPC-5=COMPLETE%22%20%22blaKPC-18=COMPLETE%22%20%22blaKPC-71=COMPLETE%22%20%22blaKPC-23=COMPLETE%22%20%22blaKPC-12=COMPLETE%22%20%22blaKPC-144=COMPLETE%22%20%22blaKPC-181=COMPLETE%22%20%22blaKPC-14=COMPLETE%22%20%22blaKPC-157=COMPLETE%22%20%22blaKPC-66=COMPLETE%22%20%22blaKPC-78=COMPLETE%22%20%22blaKPC-8=COMPLETE%22%20%22blaKPC-45=COMPLETE%22%20%22blaKPC-72=COMPLETE%22%20%22blaKPC-166=COMPLETE%22%20%22blaKPC-173=COMPLETE%22%20%22blaKPC-44=COMPLETE%22%20%22blaKPC-56=COMPLETE%22%20%22blaKPC-84=COMPLETE%22%20%22blaKPC-90=COMPLETE%22%20%22blaKPC-17=COMPLETE%22%20%22blaKPC-46=COMPLETE%22%20%22blaKPC-25=COMPLETE%22%20%22blaKPC-62=COMPLETE%22%20%22blaKPC-93=COMPLETE%22%20%22blaKPC-10=COMPLETE%22%20%22blaKPC-122=COMPLETE%22%20%22blaKPC-170=COMPLETE%22%20%22blaKPC-176=COMPLETE%22%20%22blaKPC-35=COMPLETE%22)%20AND%20isolation_source:(%22river%20water%22%20%22river%22%20%22urban%20river%22%20%22urban%20river%20water%22%20%22isabela%20river%22%20%22river%20downstream%20water%22%

	20%22surface%20water%20of%20the%20suquia%20river%206%20km%20downstream%20of%20wastewater%20treatment%20plant%22%20%22lake%22%20%22water%20sample%20from%20urban%20lake%22%20%22sea%22%20%22seawater%22%20%22coastal%20water%22%20%22stream%20water%22%20%22surface%20water%22)
VIM	https://www.ncbi.nlm.nih.gov/pathogens/isolates/#AMR_genotypes:(%22blaVIM-2=COMPLETE%22%20%22blaVIM-1=COMPLETE%22%20%22blaVIM-4=COMPLETE%22%20%22blaVIM-80=COMPLETE%22%20%22blaVIM=COMPLETE%22%20%22blaVIM-5=COMPLETE%22%20%22blaVIM-20=COMPLETE%22%20%22blaVIM-19=COMPLETE%22%20%22blaVIM-11=COMPLETE%22%20%22blaVIM-6=COMPLETE%22%20%22blaVIM-61=COMPLETE%22%20%22blaVIM-23=COMPLETE%22%20%22blaVIM-24=COMPLETE%22%20%22blaVIM-26=COMPLETE%22%20%22blaVIM-36=COMPLETE%22%20%22blaVIM-27=COMPLETE%22%20%22blaVIM-29=COMPLETE%22%20%22blaVIM-78=COMPLETE%22%20%22blaVIM-86=COMPLETE%22%20%22blaVIM-33=COMPLETE%22%20%22blaVIM-75=COMPLETE%22%20%22blaVIM-3=COMPLETE%22%20%22blaVIM-47=COMPLETE%22%20%22blaVIM-95=COMPLETE%22%20%22blaVIM-60=COMPLETE%22%20%22blaVIM-7=COMPLETE%22%20%22blaVIM-12=COMPLETE%22%20%22blaVIM-28=COMPLETE%22)%20AND%20isolation_source:(%22river%22%20%22river%20water%22%20%22sea%22%20%22surface%20water%22%20%22environmental%20sample%20water%20no.%205%22)
VCC	https://www.ncbi.nlm.nih.gov/pathogens/isolates/#AMR_genotypes:(%22blaVCC-1=COMPLETE%22%20%22blaVCC=COMPLETE%22)%20AND%20isolation_source:(%22surface%20water,%20bathing%20site%22)
blaL	https://www.ncbi.nlm.nih.gov/pathogens/isolates/#AMR_genotypes:(%22blaL1=COMPLETE%22%20%22blaL2=COMPLETE%22)%20AND%20isolation_source:(%22freshwater%20stream%22)
FRI	https://www.ncbi.nlm.nih.gov/pathogens/isolates/#AMR_genotypes:(%22blaFRI=COMPLETE%22%20%22blaFRI-8=COMPLETE%22%20%22blaFRI-9=COMPLETE%22%20%22blaFRI-10=COMPLETE%22%20%22blaFRI-11=COMPLETE%22%20%22blaFRI-4=COMPLETE%22%20%22blaFRI-2=COMPLETE%22%20%22blaFRI-5=COMPLETE%22)%20AND%20isolation_source:(%22lake%20water%22)
IMI	https://www.ncbi.nlm.nih.gov/pathogens/isolates/#AMR_genotypes:(%22blaIMI-1=COMPLETE%22%20%22blaIMI-2=COMPLETE%22%20%22blaIMI-6=COMPLETE%22%20%22blaIMI-9=COMPLETE%22%20%22blaIMI-4=COMPLETE%22%20%22blaIMI=COMPLETE%22%20%22blaIMI-12=COMPLETE%22%20%22blaIMI-16=COMPLETE%22%20%22blaIMI-29=COMPLETE%22%20%22blaIMI-17=COMPLETE%22%20%22blaIMI-20=COMPLETE%22%20%22blaIMI-25=COMPLETE%22%20%22blaIMI-13=COMPLETE%22%20%22blaIMI-10=COMPLETE%22%20%22blaIMI-19=COMPLETE%22%20%22blaIMI-24=COMPLETE%22%20%22blaIMI-26=COMPLETE%22%20%22blaIMI-8=COMPLETE%22)%20AND%20isolation_source:(%22seawater%22%20%22natural%20water%20sources%20close%20by%20farm%20or%20community%22%20%22stream%20water%22%20%22natural%20water%20sources%20by%20community%20and%20farm%22)
GES	https://www.ncbi.nlm.nih.gov/pathogens/isolates/#AMR_genotypes:(%22blaGES-5=COMPLETE%22%20%22blaGES-4=COMPLETE%22%20%22blaGES-14=COMPLETE%22%20%22blaGES-20=COMPLETE%22%20%22blaGES-24=COMPLETE%22%20%22blaGES-40=COMPLETE%22%20%22blaGES-50=COMPLETE%22%20%22blaGES-47=COMPLETE%22%20%22blaGES-54=COMPLETE%22%20%22blaGES-

	66=COMPLETE%22%20%22blaGES-58=COMPLETE%22)%20AND%20isolation_source:(%22lake%20water%22%20%22river%20water%22)
CphA	https://www.ncbi.nlm.nih.gov/pathogens/isolates/#https://www.ncbi.nlm.nih.gov/pathogens/isolates/#AMR_genotypes:(%22cphA=COMPLETE%22%20%22cphA-16=COMPLETE%22%20%22cphA-14=COMPLETE%22)%20AND%20isolation_source:(%22river%20water%22%20%22river%20don%22%20%22waterfall%22%22%22aquatic%22)%20)
OXA	https://www.ncbi.nlm.nih.gov/pathogens/isolates/#AMR_genotypes:("blaOXA-23=COMPLETE" "blaOXA-24=COMPLETE" "blaOXA-48=COMPLETE" "blaOXA-51=COMPLETE" "blaOXA-58=COMPLETE" "blaOXA-65=COMPLETE" "blaOXA-66=COMPLETE" "blaOXA-69=COMPLETE" "blaOXA-72=COMPLETE" "blaOXA-69=COMPLETE" "blaOXA-64=COMPLETE" "blaOXA-65=COMPLETE" "blaOXA-71=COMPLETE" "blaOXA-68=COMPLETE" "blaOXA-143=COMPLETE" "blaOXA-181=COMPLETE" "blaOXA-214=COMPLETE" "blaOXA-231=COMPLETE" "blaOXA-270=COMPLETE" "blaOXA-286=COMPLETE" "blaOXA-372=COMPLETE" "blaOXA-679=COMPLETE") AND isolation_source:("river water" "river" "isabela river" "river estuary" "seine river" "water sample of tiete river" "surface water" "seawater" "lake water")
